# Bridging beauty and wellness: examining beauty professionals’ comfort in discussing sexual health

**DOI:** 10.3389/frph.2025.1688125

**Published:** 2026-01-09

**Authors:** Stephanie Villaire, Savannah Hastings, McKynzie Johnson, Quinceola Reid, Sakina Ghafoor, Pamela D. Carey, Randi P. Proffitt, Kevin Knight

**Affiliations:** Institute of Behavioral Research, Texas Christian University, Fort Worth, TX, United States

**Keywords:** beauty shop model, health communication, HIV prevention, sexual health, STI prevention

## Abstract

**Background:**

Gaps in sexual health service uptake and education disproportionately effect Black women in the United States. Education is crucial to increasing health service uptake, and new avenues to provide education are continuously being developed. This current study examines beauty professionals’ comfort in discussing important women's health topics with their clients and explores their potential role as community health advocates.

**Methods:**

A total of 38 beauty professionals in Tarrant and Dallas counties, Texas completed a needs assessment survey. Descriptive statistics, *t*-tests, and correlation analyses were used to explore levels of comfort and frequency in discussing health topics, factors influencing willingness to engage in these conversations, and perceived barriers within beauty shop settings.

**Results:**

Beauty professionals reported significantly greater comfort in discussing health topics than the actual frequency with which these conversations occur. Key factors associated with increased engagement included comfort with sharing health information and the perceived importance of clients’ access to healthcare services. Identified barriers included stigma, concerns about privacy, and a lack of confidence or training in providing health information.

**Conclusions:**

Findings highlight several factors that may inhibit health-related conversations in beauty settings. The results underscore the potential for targeted interventions to support beauty professionals as trusted health messengers within their communities.

## 1 Introduction

The importance of the need for sexual health education is evidenced by high rates of new sexually transmitted infections (STIs), particularly in North Texas (1, 2). Black women experience disproportionately higher rates of STIs including chlamydia, gonorrhea, syphilis, and the human immunodeficiency virus (HIV) compared to their White counterparts. Transmission rates for HIV and STIs among Black women are up to 6.9 times higher compared to White women (3). Black women are also less likely to access preventative services including cancer screenings and contraception (4, 5). A lack of sexual health education, including school-based sexual education, can lead to negative or inaccurate information about sexual health and wellness (6). Research examining this disparity and methods to overcome it are essential to eliminating this gap in health education and service uptake.

Among Black women, there are many known barriers to sexual health service and prevention uptake including medical mistrust, stigma, misconceptions about preventative medications like pre-exposure prophylaxis (PrEP), and lack of education (7, 8). Social determinants of health, such as transportation and socioeconomic status, also play a significant role (9, 10). There is also a correlation between substance use before or during sex and risk of contracting an STI (11). Additionally, many perceive their HIV and STI risk as low (12–14). A primary barrier to PrEP uptake among Black women is a lack of understanding about their HIV risk. Ojikutu and colleagues (2020) demonstrated a disconnect between actual and self-assessed HIV risk such that the perception of one's risk is lower than their actual risk (15). Not only is healthcare access limited among Black Americans, such as a lack of insurance coverage (16), but retention in such services and medical adherence remains low due to experiences of racism resulting in medical mistrust (17). As a result, in 2020 Black Americans showed the lowest rate of receiving and remaining in HIV care compared to other ethnic groups (18, 19).

Previous research has implemented a barbershop/beauty shop model to provide health education to clients (20, 21). Beauty shops have been demonstrated to be “community anchors,” especially among Black neighborhoods, describing beauty professionals as “life counselors” (22). The length of appointments and the natural rapport beauty professionals build with clients creates an atmosphere conducive to providing health information (23, 24). As trusted community members, beauty professionals have a unique ability to discuss potentially difficult and personal topics with clients. To do this, however, beauty professionals must first be comfortable having such conversations and have correct information to disseminate. The beauty shop model trains barbers and beauty professionals to deliver healthcare information to their clients to increase health service engagement. Understanding that education is not the sole barrier to healthcare uptake, interventions such as mobile health services can play a vital role in overcoming barriers and may enhance sexual health service uptake among Black women. Mobile health services have successfully been used to provide healthcare to underserved or vulnerable populations to reduce healthcare barriers (25, 26).

The current study's parent project, the Beauty Shop Study, is a pilot that aimed to combine the beauty shop model with mobile health services to get Black women connected to sexual health services including pap smears, PrEP prescriptions, and breast exams. The Beauty Shop Study adapted the well-established beauty shop model and integrated it with mobile health service delivery to reduce common barriers to care such as limited clinic access, lack of transportation, and mistrust of the healthcare system.

Beauty professionals served as community health advocates, receiving training on how to discuss women's health, sexual health, and PrEP with their clients. Beauty professionals participated in a structured training session led by research staff during a single 90-minute Zoom call. This virtual format was designed to accommodate beauty professionals' busy schedules while still providing comprehensive instruction. During the session, participants received foundational information about HIV transmission and prevention, as well as an overview of PrEP tailored specifically to women's health needs (27). The training also emphasized communication skills, including strategies for initiating and navigating sensitive conversations with clients in ways that felt natural within the salon environment. The training was a combination of information delivery via slideshow and active practice in breakout groups. After receiving training, beauty professionals engaged clients in brief conversations during routine salon appointments, offering educational materials and answering questions related to sexual and reproductive health. When clients expressed interest in receiving services, beauty professionals referred them directly to a study-operated mobile health unit. The mobile health unit provided on-site services such as Pap smears, clinical breast exams, PrEP prescriptions, and linkage to follow-up care. By positioning health services in familiar community environments and using beauty professionals as trusted messengers, the study aimed to increase uptake of sexual healthcare among Black women (27, 28).

Prior to implementing the intervention, it was essential to develop a thorough understanding of the community the researchers intended to engage. This was accomplished through a community needs assessment (NA) that allowed researchers to gain feedback from community members about current practices and potential barriers before implementing the intervention. Prior to rollout the Beauty Shop Study team conducted two activities, a NA survey and focus groups, to garner feedback from beauty professionals in Tarrant and Dallas counties in Texas. The goal of the NA survey was to get a better understanding of the culture and dynamics of the beauty shops/salons that we aimed to recruit from and understand the feasibility of the proposed intervention, particularly mobile health services. Focus groups were conducted independently of the NA survey, therefore an in-depth discussion of qualitative results will be reported in a separate manuscript. The current study utilized data from the NA survey to explore beauty professionals’ comfort in discussing sexual health topics with their clients. Specifically, we aimed to answer two research questions (RQs): (**RQ1**) how well do beauty professionals' reported comfort levels discussing health topics match their frequency of actual conversations with clients? and (**RQ2**) what are barriers to having health conversations in beauty shops?

## 2 Materials and methods

The NA survey consisted of four sections with questions pertaining to how beauty shops operate, the nature of beauty professional-client interactions, and feedback on the use of mobile health services to serve clients in a convenient way nearby beauty shops. The NA survey was developed by the research team, tailored to the Beauty Shop Study protocol. Considering the specific purpose of the NA survey to understand attitudes towards health conversations among beauty professionals, the research team, supported by the community advisory board, identified four main topic areas of interest: Shop Basics and Atmosphere, Health Conversations, Implementation, and Demographics. *Shop Basics and Atmosphere* collected shop specific information like location, structural set-up, and details on the services provided (e.g., “How long is a typical session with one client?” and “What is the main way clients are referred to you?”). Generalized information on client relationships, patronage trends, and the main source of clients was collected. *Health Conversations* reviewed how comfortable beauty professionals are with discussing certain health topics with their clients and how often they discuss them (e.g., “Please select all health services you have ever recommended to a client” and “To what extent do you think stigma reduces your clients’ willingness to discuss women's health?”). Topics included general women's health, interpersonal relationships, family planning, and mental health. The survey also assessed how COVID-19 impacted their operations and their thoughts on medical mistrust. *Implementation* focused on what beauty professionals perceived as barriers to having health conversations with their clients as well as barriers for clients to access both community and mobile healthcare resources (e.g., “What do you perceive as barriers to increasing health conversations between you and your clients?” and “What do you perceive as barriers to have a mobile health unit provide women's health services to clients in your community?”). *Demographics* collected personal demographics of beauty professionals as well as their employment information such as licensure, time in the field, and practiced specialty.

This study used a cross-sectional, observational design in which beauty professionals completed a self-administered needs assessment survey. The study was conducted in community-based settings, including beauty industry expos, partner organization events, and online platforms available to participants across Tarrant and Dallas counties. Beauty professionals were recruited through community outreach events and word-of-mouth referrals between May and September of 2024. Community outreach events were promoted through partner organizations and the project's social media pages. These events included panel discussions with guest speakers active in the beauty and/or healthcare industries as well as beauty industry expo events. Interested beauty professionals signed up through an online interest form via a QR code or directly with project staff. Interested participants then completed informed consent and the NA survey. Beauty professionals were eligible to participate if they were 18 years of age or older, worked in Tarrant or Dallas counties, and worked in the beauty field (e.g., cosmetologists, aestheticians, waxers, nail techs, etc.). The research team aimed to recruit 40 beauty professionals across the two counties to reach a sample that was diverse and informative enough to identify common needs and patterns. Ultimately, 38 beauty professionals completed the NA survey.

### 2.1 Research team perspective

In line with recommendations from Roberts and colleagues (2020), researchers should consider how their own identities may shape the work they produce (29, 30). As such, the research team membership was intentionally constructed to be diverse, representing both females and males, as well as multiple races and ethnicities. Although none of the research team members involved in the data collection were beauty professionals or had prior experience working in in the beauty industry, the study design and implementation were guided by a diverse community advisory board comprised of mostly Black women researchers, medical professionals, experts in HIV health education, social workers, and current and former beauty professionals. Along with two paid consultants with experience in barbershop intervention studies, the community advisory board provided ongoing feedback and suggestions about how to best develop the intervention, recruit beauty professionals to partner with the study, and recruit research participants. In addition, due to well-documented experiences of medical mistrust for Black individuals, study leadership specifically sought out an experienced women's healthcare provider who identified as a Black woman to staff the mobile health unit to ensure maximum comfortability.

### 2.2 Data analysis

In total, 38 beauty professionals completed the NA survey. Participants were an average age of 35.9 years old, had an average time working in the beauty industry of 11.9 years, and had an average time working in their current shop of 3.2 years (see Table 1 for demographics).

**Table 1:** Demographic characteristics of participants (*n* = 38).

Demographic characteristic	*n*	%
Gender		
Female	32	84
Male	1	3
Not reported	5	13
Race		
Black/African American	30	79
White	2	5
Other	1	3
Not reported	5	13
Ethnicity		
Not Hispanic	29	76
Hispanic	4	11
Not reported	5	13
Currently or previously certified or licensed	29	76
Services offered^a^		
Haircut	22	58
Braiding	25	66
Hair coloring	19	50
Sew in weaves	20	53
Hair treatments	20	53
Extensions	18	47
Blow outs	21	55
Hair relaxer treatment	14	37
Wig cutting/styling	14	37
Special occasion hair styling	15	40
Silk press	20	53
Twists	23	61
Waxing	12	32
Eyebrow treatments	8	21
Eyelash extensions	11	29
Manicures/pedicures	7	18
Massages	3	8
Makeup/facials	13	34

a Participants could select multiple services offered.

The NA survey data was analyzed with paired samples *t*-tests, correlations, and frequencies. The paired samples *t*-tests were conducted to compare the responses to the questions “how *comfortable* do you feel discussing these topics” and “how *often* do you discuss these topics” on a 5-point Likert scale. We recorded the frequencies of specific barriers to health conversations and evaluated the correlations between the importance of clients' access to sexual health services and the discussion of safe sex practices.

## 3 Results

### 3.1 RQ1: comfort discussing vs. actual conversations

To understand the difference in reported comfort discussing health-related topics and how often beauty professionals actually have these conversations, paired samples *t*-tests were run. Specifically, comparisons were run between the questions “how *comfortable* do you feel discussing these topics” and “how *often* do you discuss these topics” as they relate to women's health, STIs including HIV, safe sex practices, pap smears, and birth control. The phrasing of the question does not specify if the client or the beauty professional is initiating the conversation, therefore only capturing the frequency of conversations. Both questions were answered on a 5-point Likert scale. All comparisons were significant (*p*s < .001; see Table 2 for statistics). For each parameter, beauty professionals reported being significantly more comfortable discussing these topics than their frequency of having health-related conversations.

**Table 2:** Results of comparisons between comfort discussing and frequency of actual conversations (*n* = 38).

Parameter	How often		Comfort		*t*	df	*p*	Cohens *d*
	*M*	SD	*M*	SD				
Womens health	3.50	1.11	4.32	0.82	−4.62	35	<.001	1.08
STIs	2.46	1.19	3.89	1.13	−6.38	36	<.001	1.37
Safe sex practices	2.81	1.08	4.24	1.04	−8.36	36	<.001	1.04
Pap smears	2.57	1.12	4.03	1.21	−1.04	36	<.001	1.26
Birth control	2.95	1.15	4.17	1.18	−5.89	35	<.001	1.25

To further understand beauty professionals’ attitudes toward having health-related conversations with their clients, Spearman's correlations were calculated between a set of topical questions. These questions are: (1) how often do you discuss safe sex practices with clients? (2) how comfortable do you feel discussing safe sex practices with clients? (3) how important is knowing your clients have access to sexual health services? and (4) to what extent do you think stigma reduces your clients' willingness to discuss safe sex practices? Results revealed several significant correlations (see Table 3). The frequency of discussing safe sex was positively correlated with comfort discussing safe sex (*ρ* = .449, *p* = .005), such that the more comfortable beauty professionals are discussing safe sex, the more often they discuss it. Higher stigma scores were also positively correlated with the importance of knowing clients have access to care (*ρ* = .410, *p* = .013).

**Table 3:** Correlation matrix for health conversation attitudes (*n* = 38).

Question	1	2	3	4
1. How often do you discuss safe sex practices with clients?	–	.449**	.268	.233
2. How comfortable do you feel discussing safe sex practices with clients?		–	.307	.226
3. How important to you is knowing your clients have access to sexual health services?			–	.410*
4. To what extent do you think stigma reduces your clients willingness to discuss safe sex practices?				–

** *p* < 0.01; *p < .05.

### 3.2 RQ2: barriers to health conversations

Beauty professionals were asked about their perceived barriers to increasing the frequency of health conversations with their clients. Participants were given a list of potential barriers and asked to select all that apply to their work, as well as an open-ended “other” option. The two “other” responses recorded are paraphrased in Table 4. The most reported barriers were the beauty professional not feeling equipped to share health information (64%), stigma (58%), not wanting to get too personal (58%), judgement from peers (55%), and concern about privacy (52%; see Table 4).

**Table 4:** Barriers to increasing health conversations between beauty professionals and clients (*n* = 38).

Barrier	*n*	%
Stigma	19	58
Judgement from peers	18	55
Concern about confidentiality/privacy	17	52
Lack of beauty professional knowledge about resources	16	48
Beauty professional not feeling equipped to share information	21	64
Fear of damaging relationship	9	27
Appointment time not long enough	5	15
Not wanting to get too personal	19	58
Outside of scope of my job	1	3
No barriers	1	3

## 4 Discussion

This study highlights the potential for beauty professionals to serve as effective community advocates for women's health while also highlighting key barriers to implementation. Findings from the NA survey reveal that although beauty professionals report a high level of comfort discussing health topics, these conversations occur less frequently in practice. Major barriers identified by beauty professionals include stigma, concern about privacy, and a lack of knowledge or training—indicating a need for targeted education to empower beauty professionals to initiate and navigate these discussions confidently (21, 31–33). These barriers are particularly relevant in the context of women's health, where topics such as contraception, STIs, and cancer screenings are often considered highly personal and stigmatized. For many Black women, the lack of accessible, trustworthy health information means that beauty professionals could be among the few trusted figures with whom they feel comfortable discussing reproductive health, birth control, or routine screenings. Without adequate training and support, however, opportunities to connect clients to preventive care may be missed.

Importantly, beauty professionals who expressed greater comfort discussing sensitive topics and placed a high value on client access to sexual health services were more likely to report engaging in these conversations. These findings point to a clear path forward: providing education and structured training to beauty professionals may be a viable method of increasing health conversations and referrals, specifically for women's and sexual health. Training programs could cover not only HIV and STI prevention but also broader preventive services such as pap smears, mammograms, contraceptive counseling, and safe sex practices. By equipping beauty professionals with accurate, culturally sensitive information and clear referral pathways to mobile or community-based providers, interventions can normalize conversations about women's health in everyday settings. This dual approach of enhancing comfort and knowledge among beauty professionals while reducing structural barriers through mobile services is crucial for addressing long-standing disparities in sexual and reproductive health among Black women. Incorporating the training model developed through the beauty shop pilot study into cosmetology and barbering education programs presents a promising strategy for expanding the reach and impact of health promotion efforts. By partnering with state certification boards and local cosmetology schools, health education—particularly focused on sexual health—can be integrated into existing curricula. This approach would equip future beauty professionals with the knowledge and confidence to engage in personal health-related conversations with clients and facilitate referrals to appropriate health services, thereby improving access to care among women of color. Given that safety and hygiene training is already a mandated component of licensure, embedding this content into initial instruction or periodic refresher courses offers a feasible and sustainable pathway for implementation.

A foundational step toward implementing this curriculum enhancement involves engaging with local cosmetology schools and beauty professionals to better understand their existing training frameworks and any limitations therein. These stakeholders are uniquely positioned to facilitate connections with licensing and certification boards, making them essential partners in advancing this initiative. By requesting opportunities to present at board meetings, proponents of the curriculum can introduce the concept as a valuable addition to current training standards—one that not only reinforces safe practices among beauty professionals but also extends those practices to include client well-being through health education and referral support.

Findings from the current study can be better understood in the context of the information-motivation-behavioral skills (IMB) model (34). This model focuses on the relationship between the information a person has about a specific health behavior, the individual's attitudes toward that behavior, and the individual's ability to perform the behavior. In the case of the NA survey, the goal is to understand how to leverage beauty professionals to increase the health information a person has at their disposal while motivating a person to access women's health services. According to the IMB model, this should in turn increase uptake of women's and sexual health services among clients who receive health education from their beauty professionals (e.g., 31). Future studies, including the parent study, can focus on the effects of the beauty shop education model and understanding its effect on health behaviors. This includes measuring the efficacy of the training the study provided to beauty professionals and tracking related health outcomes such as referrals to health services, instances of health-related conversations during beauty appointments, and confidence discussing health concerns with clients.

Additionally, the study offered valuable insight into the feasibility, acceptability, and implementation of the parent intervention aimed at training beauty professionals to become community health advocates, engage clients in women's health and sexual health education, and provide referral to the study's mobile health unit. After the NA identified barriers to health conversations in beauty shops, the study team modified the training for beauty professionals to include explicit conversations about stigma and privacy. For example, the modified training allowed beauty professionals to practice talking about women's health with clients at various levels of specificity. Some clients may want to talk more generally about check-ups and regular pap smears, but not as much about STIs and condom use. Other clients may be comfortable discussing these more sensitive topics and the beauty professional can provide more in-depth information about HIV risk and PrEP. The study team also created a variety of posters and handouts for beauty professionals to have in their shops. These materials had QR codes linking to web-based health information, allowing clients to read more about topics they may be uncomfortable discussing in person. These posters and handouts also served as a recruitment tool, allowing clients to contact the study team via one of the QR codes.

These findings contribute to the growing literature on community-based health promotion and align with successful beauty shop models. The results support the use of beauty shops as trusted community spaces for disseminating health information. Future research should explore how to integrate sexual and overall wellness education into beauty professionals' training programs and assess long-term impacts on client health outcomes. Future work can also aim to clarify who is more likely to bring up health-related topics—clients or beauty professionals. This distinction is key in providing focused training on either responding to clients when they initiate conversations or how to bring up health conversations when it does not tend to be discussed by clients. Other applications include training beauty professionals or other trusted community members to share information about substance use and addiction, including its intersection with impaired decision making and risky sexual behaviors. Using substances just before or during sex can increase a person's risk of having unprotected sex and contracting an STI, underscoring the importance of prevention efforts like PrEP (9, 26).

These findings are consistent with prior research demonstrating the feasibility of using beauty shops to reach populations at risk of health disparities (21, 35). Beauty shops are often trusted and familiar spaces where clients engage in open and personal conversations with their stylists, providing unique opportunities to share health information in a comfortable and non-clinical environment. A qualitative study conducted by Johnson and colleagues (36, 37) showed that client trust in beauty professionals was conducive for introducing PrEP education in beauty salons. Their work suggests that beauty professionals can act as influential messengers, helping to increase awareness, reduce stigma, and facilitate access to HIV prevention resources among clients who may not otherwise be reached through traditional healthcare systems.

Taken together, these findings emphasize that beauty shops are trusted community institutions that can bridge critical gaps in women's health. By integrating sexual and reproductive health education into these settings, beauty professionals can play a pivotal role in connecting Black women to preventive services that they might not otherwise access. Importantly, this approach focuses on meeting women where they are, both physically and culturally. Combining beauty shop education with mobile health services has the potential to increase uptake of screenings, contraception, and HIV prevention, while also boosting overall trust in healthcare. As public health systems work towards decreasing new STI and HIV diagnoses, particularly among Black women, leveraging beauty shops as health-promoting spaces offers an innovative, culturally grounded path toward advancing equity.

### 4.1 Limitations

This study has limitations that are important to acknowledge. First, the sample was small and lacked geographic diversity. All respondents were beauty professionals based in Dallas and Tarrant counties, which each have their own unique economic, social, and cultural characteristics which may not reflect the overall experiences or attitudes of beauty professionals in other parts of Texas or across the country. The small sample size also limits the power of any results, which is confounded by the missing demographic data. Additionally, the setting in which data was collected may have influenced the quality of the responses. Surveys were often conducted at busy events such as expos and social gatherings, where there was limited time for participants to engage thoughtfully. Finally, there is a potential self-selection bias where beauty professionals who are more inclined to have health conversations or who prioritize health education may have been more likely to complete the survey.

## 5 Conclusion

The NA survey provided important insights into beauty professionals' opinions on having health conversations within their practices and sets the groundwork for additional interventions aimed at expanding beauty professionals’ conversations to include other health-related topics such as substance use. While many reported feeling comfortable talking about health topics, implementation challenges like not having enough time, training, or clarity about what is appropriate to discuss often get in the way. By working with the trust that already exists in beauty service environments, we can continue to engage in interventions that are community-centered to promote health and wellness. Continued research and support for programs like the Beauty Shop Study can make a difference in reaching underserved communities and closing gaps in healthcare access.

## Data Availability

The raw data supporting the conclusions of this article will be made available by the authors, without undue reservation.
